# Methanogenic Community Was Stable in Two Contrasting Freshwater Marshes Exposed to Elevated Atmospheric CO_2_

**DOI:** 10.3389/fmicb.2017.00932

**Published:** 2017-05-24

**Authors:** Yongxin Lin, Deyan Liu, Junji Yuan, Guiping Ye, Weixin Ding

**Affiliations:** ^1^State Key Laboratory of Soil and Sustainable Agriculture, Institute of Soil Science, Chinese Academy of SciencesNanjing, China; ^2^University of the Chinese Academy of SciencesBeijing, China

**Keywords:** bulk soil, elevated CO_2_, freshwater marsh, methanogen, rhizosphere, roots

## Abstract

The effects of elevated atmospheric CO_2_ concentration on soil microbial communities have been previously recorded. However, limited information is available regarding the response of methanogenic communities to elevated CO_2_ in freshwater marshes. Using high-throughput sequencing and real-time quantitative PCR, we compared the abundance and community structure of methanogens in different compartments (bulk soil, rhizosphere soil, and roots) of *Calamagrostis angustifolia* and *Carex lasiocarpa* growing marshes under ambient (380 ppm) and elevated CO_2_ (700 ppm) atmospheres. *C. lasiocarpa* rhizosphere was a hotspot for potential methane production, based on the 10-fold higher abundance of the *mcr*A genes per dry weight. The two marshes and their compartments were occupied by different methanogenic communities. In the *C. lasiocarpa* marsh, archaeal family *Methanobacteriaceae*, Rice Cluster II, and *Methanosaetaceae* co-dominated in the bulk soil, while *Methanobacteriaceae* was the exclusively dominant methanogen in the rhizosphere soil and roots. Families *Methanosarcinaceae* and *Methanocellaceae* dominated in the bulk soil of *C. angustifolia* marsh. Conversely, *Methanosarcinaceae* and *Methanocellaceae* together with *Methanobacteriaceae* dominated in the rhizosphere soil and roots, respectively, in the *C. angustifolia* marsh. Elevated atmospheric CO_2_ increased plant photosynthesis and belowground biomass of *C. lasiocarpa* and *C. angustifolia* marshes. However, it did not significantly change the abundance (based on *mcr*A qPCR), diversity, or community structure (based on high-throughput sequencing) of methanogens in any of the compartments, irrespective of plant type. Our findings suggest that the population and species of the dominant methanogens had weak responses to elevated atmospheric CO_2_. However, minor changes in specific methanogenic taxa occurred under elevated atmospheric CO_2_. Despite minor changes, methanogenic communities in different compartments of two contrasting freshwater marshes were rather stable under elevated atmospheric CO_2_.

## Introduction

Atmospheric CO_2_ concentration has increased dramatically over the past 250 years, and is expected to continue to rise in the future (Denman et al., 2007). Elevated atmospheric CO_2_ concentrations are predicted to affect plant growth and alter soil properties. For example, atmospheric CO_2_ enrichment has been found to increase plant photosynthesis (Sasaki et al., 2005) and biomass (Hungate et al., 1997). These changes subsequently regulate the quality and quantity of the labile organic compounds that are secreted (Drigo et al., 2008) and influence downstream consumers. Wetland ecosystems play a pivotal role in global biogeochemical cycles and are a major source of atmospheric methane (CH_4_), having contributed 32% of the global annual CH_4_ emissions in the 2000s (International Panel on Climate Change [IPCC], 2013). Elevated CO_2_ can increase the carbon supply to wetland sediment (Freeman et al., 2004a), which in turn may increase CH_4_ emissions from wetlands (Vann and Patrick Megonigal, 2003; van Groenigen et al., 2011). Thus, understanding the belowground process response to elevated CO_2_ in a wetland ecosystem may help to accurately evaluate ecosystem feedbacks to global climate change.

Freshwater marshes are a substantial source of atmospheric CH_4_, with high global CH_4_ emission rates (Ding and Cai, 2007; Flury et al., 2010). For example, although freshwater marshes only account for 27% of the total natural wetland area in China, they contribute up to 66% of the annual natural wetland CH_4_ emissions (Ding et al., 2004). *Calamagrostis angustifolia* and *Carex lasiocarpa* are two dominant plants in a freshwater marsh, but with different standing water depths on the Sanjiang plain, China. The methanogenic substrates in the *C. lasiocarpa* marsh have been identified to mainly derive from plant litter, and from root exudates in the *C. angustifolia* marsh (Lin et al., 2015). A previous study has shown that dominant methanogenic communities in the bulk soil of *C. lasiocarpa* and *C. angustifolia* marshes are clearly different (Liu et al., 2012a). It is not clear if and how methanogenic communities in the rhizosphere and roots in the *C. lasiocarpa* and *C. angustifolia* marshes respond to elevated CO_2_.

Methanogenic archaea belong to the phylum Euryarchaeota (Garcia et al., 2000; Liu and Whitman, 2008), and there are generally three types in a wetland ecosystem. Members of order *Methanomicrobiales, Methanobacteriales*, and *Methanocellales* (Rice Cluster I) have been identified to exclusively use H_2_/CO_2_ as a substrate for CH_4_ production, and are known as hydrogenotrophic methanogens (Conrad, 2007). *Methanosaetaceae* within the order *Methanosarcinales* use acetate as an energy substrate, and are named acetoclastic methanogens (Liu and Whitman, 2008). In addition, *Methanosarcinaceae* within the order of *Methanosarcinales* use H_2_/CO_2_, acetate, and methyl compounds, and are ascribed to metabolically versatile methanogens (Thauer et al., 2008). Different habitats within wetland ecosystems may provide different niches for different types of methanogens. For example, *Methanocellales* were found to dominate in the rice rhizosphere and roots (Lu and Conrad, 2005; Lu et al., 2005; Conrad et al., 2008) while *Methanoregulaceae* (Fen Cluster) were dominant in boreal fen (Galand et al., 2002; Juottonen et al., 2005; Cadillo-Quiroz et al., 2006). Previous studies have shown that elevated atmospheric CO_2_ altered the soil microbial community (Deng et al., 2012; Drigo et al., 2013; He et al., 2014; Lee et al., 2015). It has been suggested that these changes were indirectly induced by changes in root growth, followed by changes in the quality and quantity of exudates under elevated CO_2_ (Drigo et al., 2008). Studying a temperate marsh microcosm, Lee et al. (2012) found that elevated CO_2_ concentrations increased the relative abundance of hydrogenotrophic methanogens. In contrast, other studies did not identify any apparent effect on the methanogen community (Angel et al., 2012; Liu et al., 2016). Drigo et al. (2008) found that elevated atmospheric CO_2_ did not affect the microbial community in the bulk soil, but did near the roots. Therefore, the effects of elevated CO_2_ concentration on methanogen communities are still debated. It is therefore necessary to evaluate whether and how the methanogenic community structure in the bulk soil, rhizosphere and roots will respond to elevated CO_2_ in wetland ecosystems.

In the present study, the methanogenic community structure and its response to elevated atmospheric CO_2_ was measured in the bulk soil, rhizosphere soil and roots of *C. lasiocarpa* and *C. angustifolia* marshes using high-throughput sequencing. We hypothesized that (1) there is a substantial difference in the methanogenic community structure among the different compartments, including the bulk soil, rhizosphere and roots; and (2) elevated atmospheric CO_2_ concentrations will inevitably induce higher methanogenic abundance by increasing substrate supply, including root exudates, due to an increase in plant photosynthesis, and also alter the methanogenic community composition in wetland ecosystems.

## Materials and Methods

### Experimental Site

The study site is located in the Sanjiang Mire Wetland Experimental Station, Tongjiang City, Heilongjiang Province (47°35′N, 133°31′E). The area of freshwater marsh in the test region is about 105 km^2^. The elevation is 56 m above sea level, and the mean annual precipitation is approximately 600 mm. The mean annual temperature was 1.9°C, with a range of -18.8°C in January and 20.8°C in July. The dominant vegetation in the freshwater marsh varies from *Calamagrostis angustifolia* to *Carex meyeriana*, and to *Carex lasiocarpa* as the standing water depth increases (Ding et al., 2002). Standing water depth ranged from 0 to 20 cm in the *Carex lasiocarpa* marsh, from 0 to 15 cm in the *Carex meyeriana* marsh, and from 0 to 5 cm in the *Calamagrostis angustifolia* marsh. We selected a permanently inundated *C. lasiocarpa* marsh and a summer inundated *C. angustifolia* marsh for the elevated CO_2_ experiment.

### Experimental Setup

Sixteen intact soil columns with 25-cm diameter and 30-cm depth were collected using a stainless steel sampler from each plant growing wetland on 10 May 2015. These columns were immediately transferred into specially designed PVC macrocosms (40-cm height and 25-cm inner diameter) and transported to the experimental site. The standing water depth in the *C. lasiocarpa* and *C. angustifolia* marshes was maintained at 8 and 2 cm (average standing water depth in summer), respectively, during the experimental period from May to October.

All macrocosms were divided into two groups based on measured methane emission rates and transferred into two open top chambers (OTC) in the field. The methane emission rates were nearly identical in the two groups of the same plant type marsh. The OTC chambers were constructed from a PVC frame and clear, infrared-transmissive Aclar film walls (Shanghai, China) to allow natural light exposure (>95%). The tops and bottoms of the chambers were left open to allow air circulation. One chamber received an elevated CO_2_ treatment (eCO_2_), while the other received ambient air (aCO_2_). Continuous injection of pure CO_2_ into the OTC was carried out to maintain the elevated levels of CO_2_ in the OTC at 700 ± 20 ppm. The air sample was drawn from the middle of the chamber by an automated system and the CO_2_ concentration was monitored using an infrared gas analyzer (Li-7000; Licor, Lincoln, NE, United States). A rain-diversion system was constructed over the experiment site, and automatically opened on sunny days and closed on rainy days. The chamber air temperature was automatically adjusted to ±1°C of the real air temperature by a temperature control system. At the end of the growing season in October, the aboveground biomass was determined by carefully clipping plants in each macrocosm in one group at the soil surface. The plants were then washed with distilled water and oven-dried for 24 h at 70°C to constant mass. To estimate underground plant biomass, the live roots and rhizome material were manually picked out from the soil columns and then oven-dried as mentioned above.

### Measurement of Net Photosynthetic Rate and Soil Pore Water DOC Concentration

The net photosynthetic rate (Pn) was measured on flag leaves from 09:00 to 11:00 am by a LI-6400 portable photosynthesis system (LiCor Inc., Lincoln, NE, United States). For each treatment, Pn of nine leaves was determined at each measurement and the measurement repeated twice. A Rhizon MOM pore water sampler (Rhizosphere Research Products, Wageningen, the Netherlands), which consisted of a 5 cm porous part and 20 cm tubing PVC hose, was used to collect soil pore water at 10 cm depth of marsh profile. Prior to collecting samples, approximately 5 mL of pore water were extracted and discarded and then 50 mL of pore water was collected. Dissolved organic carbon (DOC) in soil pore water was analyzed on a Shimadzu C analyzer (TOC Vcph, Shimadzu, Kyoto, Japan).

### Plant and Soil Sampling and DNA Extraction

The plants and soil in the four macrocosms of each treatment in another group were destructively sampled on 8 August 2015 when the plants showed the highest activity. The plants and soil were manually separated into three compartments; bulk soil (non-rooted soil), rhizosphere soil (soil on root surface), and root. The samples from the three compartments were collected according to Bao et al. (2014). Briefly, the near-bottom and non-rooted soil was collected and designated as bulk soil. Then, the roots with surface soils were washed before and after sonication, and all soils were pooled and centrifuged for 10 min at 10,000 ×*g* at 4°C to generate the rhizosphere soil samples. After the rhizosphere soil was removed, the roots were transferred to new 50-mL Falcon tubes containing sterilized water and centrifuged for 10 min at 10,000 ×*g* at 4°C; the pellet in this centrifugation tube was designated as the root component. All samples were stored at -80°C for no more than 2 weeks prior to molecular analysis. DNA was extracted from all samples using the Fast DNA SPIN for soil kit (BIO 178 101, Qbiogene, Carlsbad, CA, United States) according to the manufacturer’s instructions. For the root samples, the frozen tissues were ground to powder in liquid nitrogen before DNA extraction.

### Real-Time Quantitative PCR

To quantify the functional marker genes (*mcr*A) of methanogens, we performed q-PCR using a CFX96 Optical Real-Time Detection System (Bio-Rad Laboratories Inc., Hercules, CA, United States). Each reaction in the 25 μl contained the specific primer set for the methanogen: MLf (GGTGGTGTMGGATTCACACARTAYGCWACAGC) – MLr (TTCATTGCRTAGTTWGGRTAGTT) (Juottonen et al., 2006). Each reaction mixture (25 μl) consisted of 12.5 μl 1 × SYBR Premix Ex Taq (Takara, Tokyo, Japan), 0.25 μl of each primer, and 1 μl of DNA template containing approximately 1–10 ng of DNA. A negative control was always run with sterilized distilled water as the template instead of a DNA sample. The amplification was initiated by denaturation at 95°C for 2 min, followed by 35 cycles of denaturation at 95°C for 10 s, annealing at 55°C for 30 s, extension at 72°C for 30 s, and the plate was read at 80°C. The standard curves were created using 10-fold dilution series of plasmid DNA containing the *mcrA* gene from environmental samples for methanogen. A serial dilution of the DNA template was also used to assess whether the PCR was inhibited during the amplification. The amplification always resulted in a single peak, and amplification efficiencies of 106.7–108.7% were obtained with *R*^2^ values of 0.992–0.999.

### High-Throughput Sequencing and Data Analysis

For methanogens, the primer set 1106F and 1378R was used to amplify approximately 280 bp of methanogenic archaeal 16S rRNA gene fragments (Feng et al., 2013). The forward primer contained a sample-identifying barcode unique to each sample. PCR amplifications were performed in 50 μl reaction mixtures containing approximately 40 ng of template DNA, 25 μl Phusion High-Fidelity PCR Master Mix (New England Biolabs), 20.5 μl H_2_O, 0.5 μl 1% bovine serum albumin (BSA), and 0.2 μM of each primer. Thirty cycles (95°C for 45 s, 56°C for 45 s, and 72°C for 60 s) were performed with a final extension at 72°C for 7 min. PCR products were mixed in equidensity ratios. Then, the mixed PCR products were purified with the Qiagen Gel Extraction Kit (Qiagen, Germany). Sequencing libraries were generated using the TruSeq DNA PCR-Free Sample Preparation Kit (Illumina, United States) following the manufacturer’s recommendations. The library quality was assessed on the Qubit 2.0 Fluorometer (Thermo Scientific) and Agilent Bioanalyzer 2100 systems. Finally, high-throughput sequencing of the methanogenic archaeal 16S rRNA genes was carried out on the Illumina HiSeq2500 platform at the Novogene (Beijing, China), and 250 bp paired-end reads were generated.

The high-throughput sequencing data were processed using the Quantitative Insights Into Microbial Ecology (QIIME) 1.7.0-dev pipeline (Caporaso et al., 2010) using default parameters, unless otherwise indicated. In brief, low quality sequence reads were trimmed and the barcode was examined to assign the multiplexed reads to samples. Next, the sequences were compared with the reference database (Edgar et al., 2011) using the UCHIME algorithm to detect chimeric sequences, and the chimeric sequences were removed. Sequences with ≥97% similarity were assigned to the same OTUs using the Uparse software (Uparse v7.0.1001) (Edgar, 2013), and the most abundant sequence from each OTU was selected as a representative sequence for that OTU. Taxonomy was assigned to methanogenic archaeal OTUs against the GreenGene Database (DeSantis et al., 2006) using a RDP classifier (Wang et al., 2007). To remove the heterogeneity of the number of sequences per sample, the sequences were rarefied prior to the calculation of the alpha diversity statistics. Alpha diversity was assessed by calculating Chao1 (Chao and Bunge, 2002), Observed species, Phylogenetic Diversity (PD), and Shannon metrics in QIIME. All sequences have been deposited in the DNA Data Bank of Japan under the accession number DRA005365.

### Statistical Analysis

The SPSS 18.0 software package for Windows (SPSS Inc., Chicago, IL, United States) was used to perform statistical analyses. All data were expressed on the basis of the oven-dried soil mass. Before analysis of variance (ANOVA), the data were checked for normality and homogeneity of variance (Levene’s test), and were subsequently ln-transformed to meet the assumptions of the ANOVA analyses, if necessary. Statistically significant differences among treatments and compartments were determined by one-way ANOVA and least significant difference (LSD) calculations at a 5% confidence level. A Non-Metric Multidimensional Scaling (NMDS) plot was constructed from the analyzed samples based on the pairwise shared OTUs to determine the change in the community structure. Three different non-parametric analysis methods, including non-parametric multivariate analysis of variance using distance matrices (adonis), analysis of similarities (ANOSIM), and a multi-response permutation procedure (MRPP), were used to examine whether there were significant differences in the community structures between the treatments. The Bray-Curtis distance was used for the adonis, ANOSIM, and MRPP analyses. NMDS, adonis, ANOSIM, and MRPP were processed with the “vegan” package in the R software version 3.0.2.

## Results

### Plant Growth and Physiological Characteristics

**FIGURE 1:**
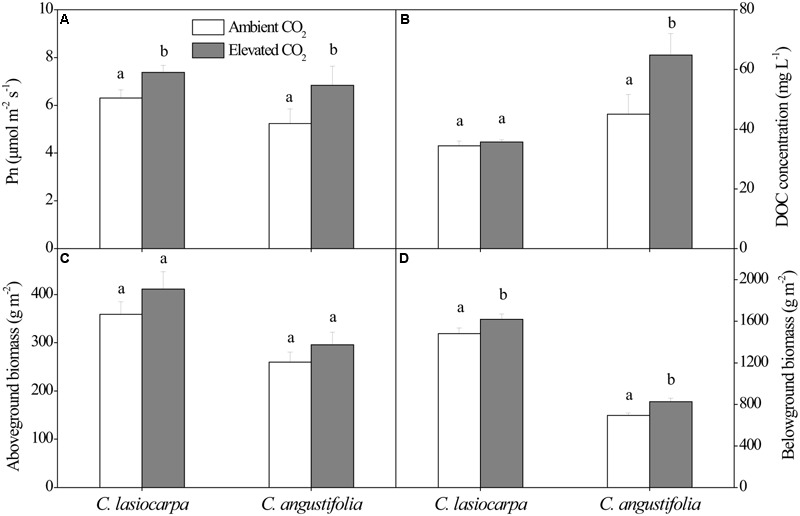
**Effect of elevated CO_2_ concentration on net photosynthetic rate (A)**, dissolved organic C (DOC) concentration in soil pore water **(B)**, aboveground biomass **(C)** and belowground biomass **(D)** in the *C. lasiocarpa* and *C. angustifolia* marshes. Different letters denote significant differences between ambient and elevated CO_2_ treatments at *P* < 0.05. Vertical bars denote the standard error of the mean (*n* = 4).

The net photosynthetic rate, DOC concentration, and aboveground and belowground biomass in the *C. lasiocarpa* and *C. angustifolia* marshes under ambient and elevated CO_2_ concentration were shown in **Figure 1**. The net photosynthetic rates in the *C. lasiocarpa* and *C. angustifolia* marshes were increased under elevated CO_2_ treatment compared with ambient CO_2_ treatment, irrespective of plant types. Elevated CO_2_ concentration increased belowground biomass in the *C. lasiocarpa* and *C. angustifolia* marshes. It also increased DOC concentration in soil pore water of *C. angustifolia* marsh.

### Abundance of Methanogenic Archaea

**FIGURE 2:**
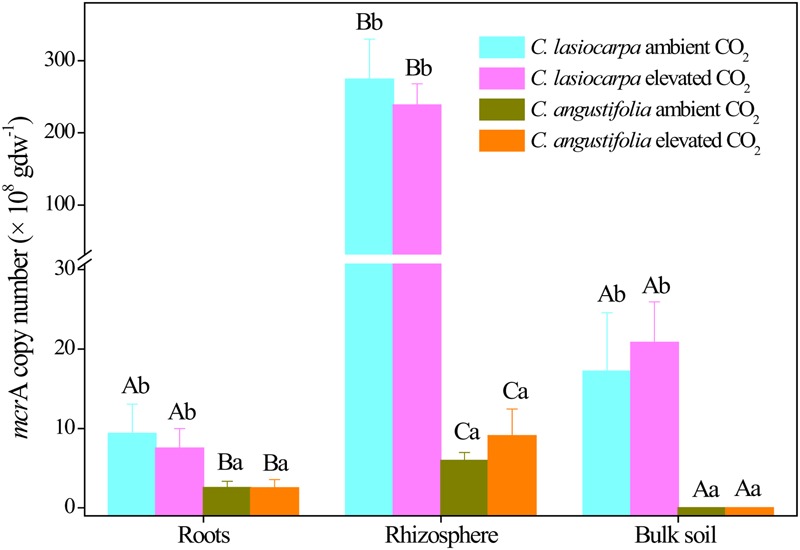
**Change in the abundances of methanogenic *mcr*A genes in the *C. lasiocarpa* and *C. angustifolia* marshes from ambient to elevated CO_2_ concentrations based on qPCR.** Different letters denote significant differences between compartments in the same treatment (capital letters) and treatments in the same compartment (lowercase letters) at *P* < 0.05. Vertical bars denote the standard error of the mean (*n* = 4).

The copy numbers of the methanogenic archaeal *mcr*A gene varied from 1.59 ± 0.98 × 10^6^ to 2.74 ± 0.56 × 10^10^ copies per g d.w.s. There appeared to be a greater abundance of the methanogenic archaeal *mcr*A gene in the rhizosphere soil than in the bulk soil and roots, especially in the *C. lasiocarpa* marsh (**Figure 2**). The *mcr*A gene abundance in the rhizosphere soil of *C. lasiocarpa* marsh was at least one order of magnitude greater than that in the bulk soil and roots. The *mcr*A gene abundance in the *C. lasiocarpa* marsh was significantly higher than that in the *C. angustifolia* marsh in all three compartments. However, *mcr*A gene abundance did not differ between ambient and elevated CO_2_ treatments in all the compartments, irrespective of plant types.

### Diversity, Richness and Taxonomic Distribution of Methanogenic Archaea

The Chao1, PD, observed species, and Shannon indices derived from high-throughput sequencing provided estimates of the microbial diversity and richness among different samples. Chao1 and observed species consistently showed that the maximum richness and diversity of methanogeic archaea in the bulk soil were much higher than those in the rhizosphere soil and roots (Supplementary Figure S1). However, there was no significant difference in methanogenic archaeal richness and diversity between rhizosphere soil and roots (**Table 1**). The elevated CO_2_ concentration reduced the PD and observed species of methanogenic archaea in the bulk soil in the *C. angustifolia* marsh, but did not affect the corresponding values in the *C. lasiocarpa* marsh. The richness and diversity of methanogenic archaea appeared similar between the elevated and ambient CO_2_ treatments in the rhizosphere soil and roots in both plant marshes.

**Table 1:** Comparison of the estimated OTU richness and diversity indices of the 16S rRNA gene libraries from the high-throughput sequencing analysis.

Plant species	Compartments	Treatments	Chao1	Phylogenetic diversity	Observed species	Shannon indices
*C. lasiocarpa*	Bulk soil	Ambient CO_2_	292.87 ± 8.02b	31.27 ± 3.47a	269.00 ± 6.03b	4.28 ± 0.19c
		Elevated CO_2_	310.54 ± 5.18b	31.86 ± 2.48a	284.67 ± 2.40c	4.52 ± 0.10c
	Rhizosphere soil	Ambient CO_2_	158.62 ± 1.99a	24.52 ± 3.86a	135.67 ± 0.33ab	2.88 ± 0.16a
		Elevated CO_2_	165.22 ± 10.28a	23.71 ± 2.00a	142.33 ± 4.98ab	2.75 ± 0.11a
	Root	Ambient CO_2_	173.06 ± 4.15a	28.52 ± 6.01a	149.33 ± 2.73b	2.95 ± 0.15a
		Elevated CO_2_	162.30 ± 6.64a	24.40 ± 2.71a	149.00 ± 2.00b	2.98 ± 0.12a
*C. angustifolia*	Bulk soil	Ambient CO_2_	539.33 ± 61.15d	232.51 ± 9.46c	429.00 ± 34.96f	4.55 ± 0.21c
		Elevated CO_2_	433.18 ± 46.51c	182.41 ± 7.93b	335.67 ± 10.91d	4.60 ± 0.10c
	Rhizosphere soil	Ambient CO_2_	121.17 ± 16.94a	28.10 ± 9.03a	109.67 ± 12.67ab	2.78 ± 0.14a
		Elevated CO_2_	118.33 ± 10.12a	28.68 ± 6.86a	106.33 ± 8.74a	3.18 ± 0.19a
	Root	Ambient CO_2_	135.58 ± 30.47a	26.75 ± 5.26a	127.33 ± 25.85ab	3.82 ± 0.19b
		Elevated CO_2_	134.25 ± 19.92a	26.58 ± 2.54a	120.33 ± 14.50ab	3.82 ± 0.14b

*Data were presented as the mean of three replicates ± standard error. Different letters within the same column indicate significant differences among treatments (*P* < 0.05). All indices were calculated using the subset of 36094 sequences per sample.*

**FIGURE 3:**
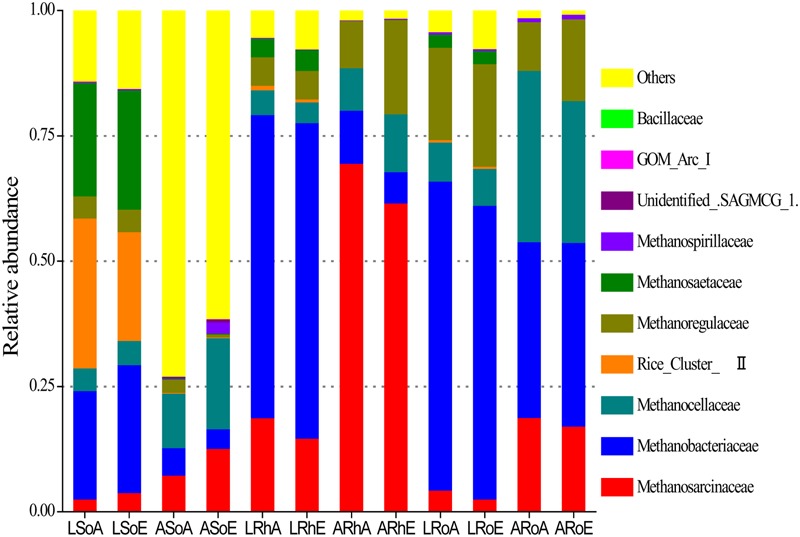
**A 100% stack column chart of the dominant methanogenic archaeal families in each sample from high-throughput sequencing.** The value of each family percentage is the mean of four replicates. Group names have been selected according to the treatments: L and A denote the *C. lasiocarpa* and *C. angustifolia* marshes, respectively; So, Rh, and Ro indicate the soil, rhizosphere soil, and root compartments, respectively; and A and E represent ambient and elevated CO_2_ concentrations, respectively.

High-throughput sequencing revealed that the methanogenic archaeal community was dominated by several families (**Figure 3**). In the bulk soil, the methanogenic archaeal community was dominated by *Methanobacteriaceae* (21.6%), Rice Cluster II (29.9%), and *Methanosaetaceae* (22.7%) in the *C. lasiocarpa* marsh; and by *Methanosarcinaceae* (7.3%) and *Methanocellaceae* (10.9%) in the *C. angustifolia* marsh. In the rhizosphere soil, the dominant methanogenic archaeal families were *Methanobacteriaceae* (60.5%) and *Methanosarcinaceae* (18.8%) in the *C. lasiocarpa* marsh; and *Methanosarcinaceae* (69.6%) and *Methanoregulaceae* (9.4%) in the *C. angustifolia* marsh. In the roots, the methanogenic archaeal community was dominated by *Methanobacteriaceae* (61.7%) and *Methanoregulaceae* (18.4%) in the *C. lasiocarpa* marsh; and by *Methanobacteriaceae* (35.1%), *Methanosarcinaceae* (18.8%), and *Methanocellaceae* (34.2%) in the *C. angustifolia* marsh. A significant difference was found in the methanogenic archaeal community between the bulk soil and roots in the same plant marsh (**Table 2**). Significant differences also occurred in the methanogenic archaeal community between the *C. lasiocarpa* and *C. angustifolia* marshes. However, there was only a small, and insignificant, variation in the methanogenic archaeal community between the elevated and ambient CO_2_ treatments in the two tested marshes.

**Table 2:** Significance tests of the effects of the CO_2_, soil or plant compartments and plant species on the overall methanogenic community structure with three statistical approaches.

Compared groups	Adonis^a^		ANOSIM^b^		MRPP^c^	
	*F*	*P*	*R*	*P*	*δ*	*P*
**CO_2_ effect**						
aCO_2_ vs. eCO_2_ at soil of *C. lasiocarpa*	0.790	0.500	0.125	0.300	0.202	0.300
aCO_2_ vs. eCO_2_ at soil of *C. angustifolia*	1.674	0.100	0.370	0.100	0.255	0.100
aCO_2_ vs. eCO_2_ at rhizosphere of *C. lasiocarpa*	1.707	0.100	0.037	0.400	0.125	0.100
aCO_2_ vs. eCO_2_ at rhizosphere of *C. angustifolia*	1.739	0.100	0.148	0.200	0.285	0.100
aCO_2_ vs. eCO_2_ at root of *C. lasiocarpa*	2.615	0.200	0.444	0.200	0.118	0.200
aCO_2_ vs. eCO_2_ at root of *C. angustifolia*	1.622	0.400	-0.074	0.600	0.255	0.400
**Compartment effect**						
Soil vs. rhizosphere at *C. lasiocarpa*	75.968	0.003	1.000	0.003	0.167	0.007
Soil vs. root at *C. lasiocarpa*	75.814	0.001	1.000	0.003	0.172	0.002
Rhizosphere vs. root at *C. lasiocarpa*	15.133	0.005	0.974	0.004	0.138	0.004
Soil vs. rhizosphere at *C. angustifolia*	36.624	0.001	1.000	0.005	0.285	0.005
Soil vs. root at *C. angustifolia*	37.188	0.001	1.000	0.004	0.270	0.004
Rhizosphere vs. root at *C. angustifolia*	15.707	0.001	0.989	0.005	0.286	0.003
**Plant species effect**						
*C. lasiocarpa* vs. *C. angustifolia*	29.87	0.001	0.948	0.001	0.495	0.001

*^a^Permutational multivariate analysis of variance using distance matrices. The significance was examined using *F*-tests based on sequential sums of squares from permutations of the raw data. ^b^Analysis of similarities. *R* was obtained based on the difference of the average ranks between and within groups. The significance of R was tested by permuting the grouping vector to obtain the empirical distribution of *R* under the null-model. ^c^Multi-response permutation procedure. The statistic δ indicates the overall weighted mean of the within-group means of the pairwise dissimilarities among the sampling units. The significance test indicates the fraction of permuted δ that is less than the observed δ.*

### Shift in the Composition of the Methanogenic Archaeal Community

**FIGURE 4:**
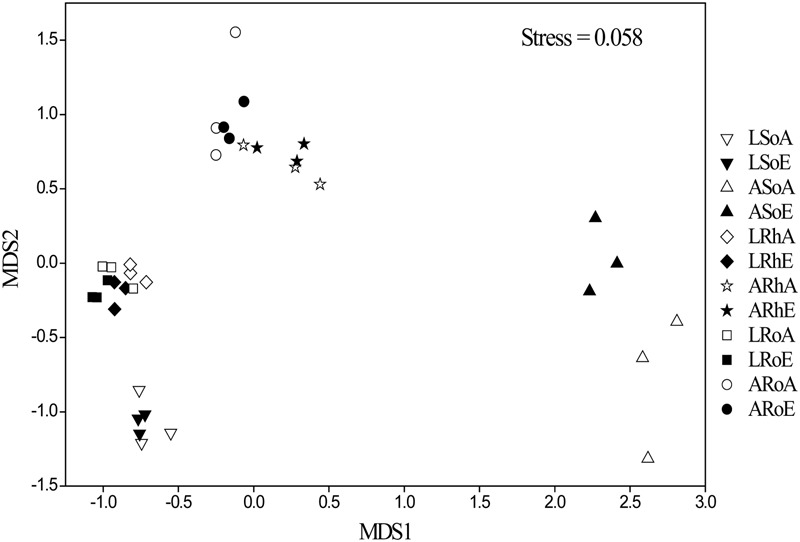
**Community composition structure of methanogenic archaea in different compartments from high-throughput sequencing, indicated by a non-metric multi-dimensional scaling plot among the different samples.** Group names have been selected according to the treatments: L and A denote the *C. lasiocarpa* and *C. angustifolia* marshes, respectively; So, Rh and Ro indicate the soil, rhizosphere soil, and root compartments, respectively; and A and E represent ambient and elevated CO_2_ concentrations, respectively.

To better visualize the shifts in the methanogenic archaeal community composition of the two plant marshes in response to elevated CO_2_, non-metric multidimensional scaling (NMDS) was performed (**Figure 4**). Elevated CO_2_ did not alter the methanogenic archaeal community composition in the bulk soil, rhizosphere soil or roots in either plant dominated marshes. This finding was further confirmed by three non-parametric multivariate analyses (adonis, ANOSIM, and MRPP). In contrast, the methanogenic communities were significantly different between the different compartments, regardless of CO_2_ levels (**Table 2**). For example, a potential shift was observed in the methanogenic archaeal community between the bulk and rhizosphere soils, irrespective of plant type. Furthermore, the methanogenic archaeal communities in the *C. lasiocarpa* marsh were grouped together and significantly separated from those in the *C. angustifolia* marsh (**Figure 4**).

### Methanogenic Archaeal Community Response to CO_2_ Enrichment

**FIGURE 5:**
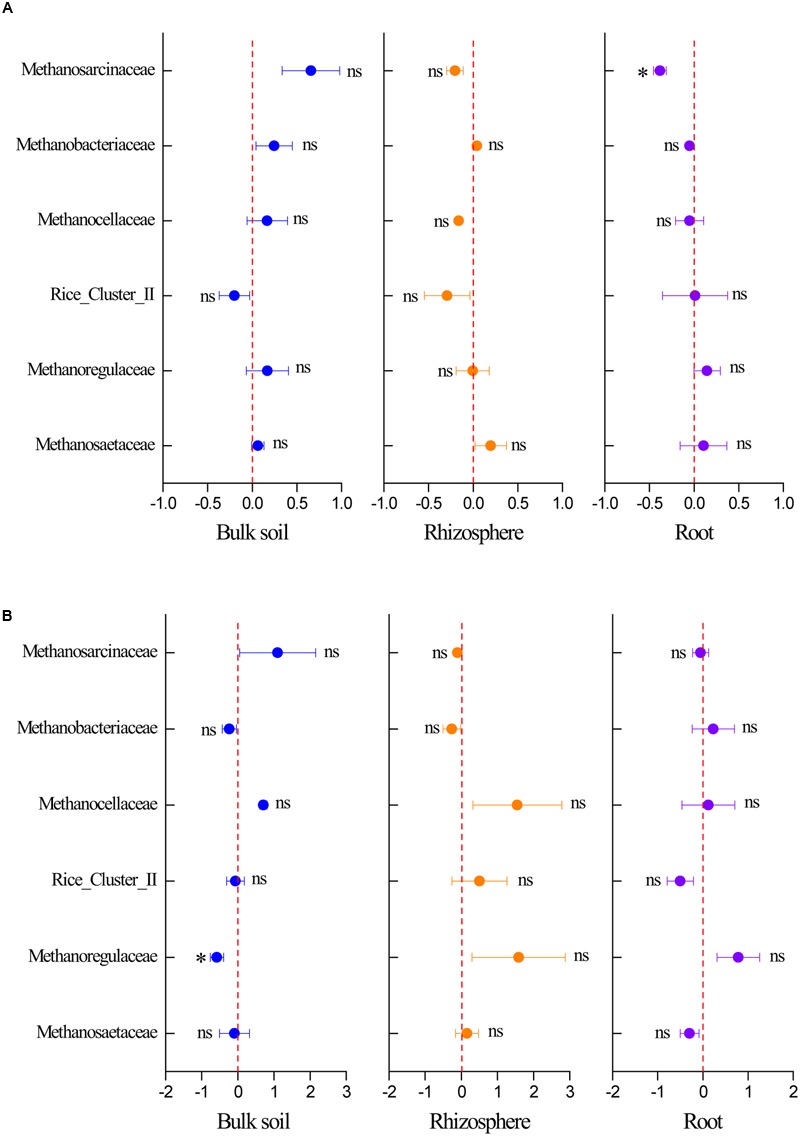
**Effect of elevated CO_2_ on the relative abundance of methanogenic taxa at the family level in the *C. lasiocarpa* (A)** and *C. angustifolia* **(B)** marshes based on high-throughput sequencing. The elevated CO_2_ effect was measured using the net difference in the relative abundance of methanogenic taxa between the elevated and ambient CO_2_ treatments and then divided by the relative abundance of the phylotype under ambient CO_2_ treatment. The error bars represent the standard error of the mean (*n* = 3). The asterisk indicates significant difference at *P* < 0.05; ns indicates no significant difference (*P* > 0.05).

The response of the methanogenic archaea to elevated CO_2_ was calculated based on their sequence numbers obtained by high-throughput sequencing to identify the respective responses of dominant methanogenic archaeal communities to elevated CO_2_. Elevated CO_2_ decreased the relative abundance of *Methanosarcinaceae* in the root compartment in the C. *lasiocarpa* marsh and *Methanoregulaceae* in the bulk soil in the *C. angustifolia* marsh (**Figure 5**). In addition, some trends were seen in the relative abundance of *Methanosarcinaceae, Methanobacteriaceae*, and *Methanosaetaceae* (**Figure 5**), but due to high variation and low sample numbers they were not statistically significant.

## Discussion

### Methanogenic Archaea in Different Niches

The composition of the methanogenic archaea community was clearly different between the soil and roots of the two plant marshes, as reported by Ke et al. (2013, 2014). In the two plant roots, *Methanobacteriaceae* were dominant although their relative abundance was lower in *C. angustifolia. Methanoregulaceae* were more abundant than *Methanocellaceae* in *C. lasiocarpa* roots and the opposite was observed in *C. angustifolia* roots, suggesting a plant-specific enrichment. This result was consistent with a previous finding in another two contrasting vascular plants that *Methanoregulaceae* were more abundant than *Methanocellaceae* on *Dulichium arundinaceum* and the opposite was observed on *Sarracenia purpurea*, in an acidic bog (Cadillo-Quiroz et al., 2010). In the *C. lasiocarpa* marsh, *Methanobacteriaceae*, Rice Cluster II, and *Methanosaetaceae* were co-dominant in the bulk soil. However, *Methanobacteriaceae* were the exclusively dominant methanogenic archaea (>60%) in the rhizosphere soil and roots. The *Methanobacteriaceae* family has been widely studied, and all species are known to use H_2_/CO_2_ as a substrate (Bonin and Boone, 2006). Previous studies have demonstrated that the specific communities of methanogens that had colonized rice roots preferred hydrogenotrophic over acetoclastic methanogens (Chin et al., 2004; Wu et al., 2009; Liu G.C. et al., 2012). *Methanocellaceae* were found to dominate particularly in the rice roots, as they have a multiple set of genes encoding for antioxidant enzymes (Erkel et al., 2006) and can produce CH_4_ even under aerobic conditions (Angel et al., 2011). In the present study, however, *Methanobacteriaceae* rather than *Methanocellaceae* dominated in the roots of *C. lasiocarpa*, which has a strong gas transport capacity delivering a lot of oxygen downward to the rhizosphere (Ding et al., 2005). Some members of the *Methanobacteriaceae* were found to survive after an exposure to oxygen (Kiener and Leisinger, 1983). Using a DNA-based stable isotope probing (DNA-SIP) technique, our previous study (Lin et al., 2015) identified *Methanobacteriaceae* as a metabolically active methanogen in the *C. lasiocarpa* marsh at the same site. These findings suggest that *Methanobacteriaceae* may play a key role in the *C. lasiocarpa* marsh. However, further study is required to evaluate the physiological characteristics and molecular mechanisms of how *Methanobacteriaceae* act in the rhizosphere and roots of the *C. lasiocarpa* marshes, which have high CH_4_ emissions (Song et al., 2009).

In the *C. angustifolia* marsh, *Methanosarcinaceae* and *Methanocellaceae* were dominant in the bulk soil, while *Methanosarcinaceae* and *Methanocellaceae* dominated in the rhizosphere soil and roots, respectively. *Methanosarcinaceae* and *Methanocellaceae* were also found to be the main community members on rice roots and associated soil (Krüger et al., 2005), and to be metabolically active methanogens in the rice roots measured by DNA-SIP (Lu et al., 2005). In addition, *Methanocellaceae* have the ability to produce CH_4_ under aerobic conditions (Angel et al., 2011) as they contain antioxidant enzymes encoded genes. It is therefore not surprising that *Methanocellaceae* dominated in the *C. angustifolia* roots. *Methanosarcinaceae* have the most versatile substrate spectrum, including H_2_/CO_2_, acetate, and methyl compounds (Thauer et al., 2008). They flourish in the rhizosphere supported by their ability to use various byproducts from root exudates and decaying plant debris (Shrestha et al., 2011). Furthermore, *Methanosarcinaceae* have the capacity to tolerate low oxygen levels and possess catalase genes that actively transcribe for oxygen detoxification (Angel et al., 2011). Consequently, *C. angustifolia* rhizosphere soil is a suitable habitat for *Methanosarcinaceae*.

### Effect of Elevated CO_2_ on Methanogenic Archaea

In this study, the difference in the *mcr*A gene abundance in the three compartments of both marshes was statistically not significant under elevated CO_2_ when compared to ambient CO_2_. In a previous paddy soil study, Liu et al. (2016) similarly did not observe an effect of elevated CO_2_ on the abundance of the methanogenic archaeal community in the bulk soil. In contrast, some studies have reported that elevated CO_2_ significantly increased the abundance of methanogenic archaea in rice cultivated soil (Inubushi et al., 2003; Okubo et al., 2015) because of the increase in the carbon supply as root exudates with elevated CO_2_ (Bhattacharyya et al., 2013). In the present study, elevated CO_2_ concentration significantly increased the plant photosynthesis and root biomass in both plant marshes (**Figure 1**) and these changes would subsequently increase C substrate for methanogens. However, the increased C substrate under elevated CO_2_ did not result in a significant increase in the abundance of methanogenic archaea (**Figure 2**). The reasons for this include: first, the increased belowground C substrate input may have not been large enough to enhance growth of the methanogenic population, or such C-input increases may have only promoted methanogenic metabolic rates rather than increased population size. The effects of elevated CO_2_ on CH_4_ emission rates in the two marshes, increasing CH_4_ emissions by 27.60% in *C. angustifolia* marsh and suppressing them by 18.67% in the *C. lasiocarpa* marsh (to be discussed in a future publication) were unrelated to responses of the methanogenic population (Lin et al., unpublished data). Secondly, the potential stimulating effect of increased substrate supply under elevated CO_2_ on the abundance of methanogenic archaea may have been counteracted by the inhibiting effect of increased delivery of O_2_ to the roots and rhizosphere (Freeman et al., 2004b). Elevated CO_2_ may enlarge aerenchyma conduits and increase root porosity and subsequently increase O_2_ concentration in the rhizosphere (Schrope et al., 1999; Cheng et al., 2000). Furthermore, the significantly increased root biomass and photosynthesis (**Figure 1**) due to elevated CO_2_ may also have more effectively aerated the soil and inhibited methanogen growth.

To date, studies have shown different responses of the soil microbial communities to elevated CO_2_. Some studies have found that elevated CO_2_ induced shifts in the microbial community composition (Lagomarsino et al., 2008; Lee et al., 2012, 2015), while others did not find any significant influence (Grüter et al., 2006; Angel et al., 2012). Gschwendtner et al. (2015) pointed out that the intensity of the effect of elevated CO_2_ on soil microbial communities was dependent on the distance from the roots. Hayden et al. (2012) and Okubo et al. (2015) suggested that elevated CO_2_ induced changes in the community structures of microorganisms mainly in the rhizosphere and roots. In the present study; however, we did not find a significant effect of elevated CO_2_ on the methanogenic community composition in the bulk soil, rhizosphere soil, or roots (**Figure 4**). The resilience of the methanogenic community to elevated CO_2_ may be due to the general robustness of the community against changes in the surrounding environment (Liu et al., 2012b, 2016), or the changes in the organic substrate input and environmental factors were potentially not large enough to change the methanogen community composition (Angel et al., 2012). Likewise, in a 10-year atmospheric CO_2_ enrichment experiment, Angel et al. (2012) did not observe changes to the methanogenic communities in a waterlogged grassland under elevated CO_2_. Therefore, it is unlikely that the lack of apparent effect of elevated CO_2_ on the methanogenic community in this study was due to the short duration of the field experimental period. Thus, we suggest that the methanogenic community was relatively stable in the two contrasting marshes even if the atmospheric CO_2_ concentration doubled. Although we did not find significant changes in the methanogenic community in response to elevated CO_2_ concentrations in either of the marshes or their compartments, minor trends were observed.

Overall, our data demonstrate that there is a clear difference in abundance and community structure of methanogens among the different compartments of two contrasting plant dominated marshes. Abundance and community structure of methanogens also differed between *C. lasiocarpa* and *C. angustifolia* marsh. *C. lasiocarpa* rhizosphere was a hotspot for potential methane production, based on the 10-fold higher abundance of the *mcr*A genes per dry weight. However, in contrast to our hypothesis, although minor difference occurred in specific methanogenic taxa in the different compartments and plant dominated marshes, the abundance and community structure of methanogens were rather stable when atmospheric CO_2_ concentrations was increased.

## Author Contributions

Conceived and designed the experiments: DL and WD. Performed the experiments: YL, JY, and GY. Analyzed the data: YL and DL. Wrote the paper: YL and WD. All authors read and approved the final manuscript.

## Conflict of Interest Statement

The authors declare that the research was conducted in the absence of any commercial or financial relationships that could be construed as a potential conflict of interest.
