# Supplementary material for: Methanogenic Community Was Stable in Two Contrasting Freshwater Marshes Exposed to Elevated Atmospheric CO2
Source: Front Microbiol. 2017 May 24;8:932. doi: 10.3389/fmicb.2017.00932 (PMC5442310; doi:10.3389/fmicb.2017.00932)
Supplement: Supplementary file 1 [file Image_1.PDF]

Methanogenic community was stable in two contrasting freshwater marshes exposed to elevated atmospheric CO_2

Authors

Yongxin Lin[1,2], Deyan Liu[1], Junji Yuan[1], Guiping Ye[1,2], Weixin Ding[1,*]

Affiliations

[1] *State Key Laboratory of Soil and Sustainable Agriculture, Institute of Soil Science, Chinese Academy of Sciences, Nanjing 210008, China*

[2] *University of the Chinese Academy of Sciences, Beijing 10049, China.*

Figure S1. Rarefaction curves of Chao1, Phylogenetic Diversity (PD) index, observed species, and Shannon index for the three compartments in the *C. lasiocarpa* and *C. angustifolia* marshes under ambient and elevated CO_2 concentrations.

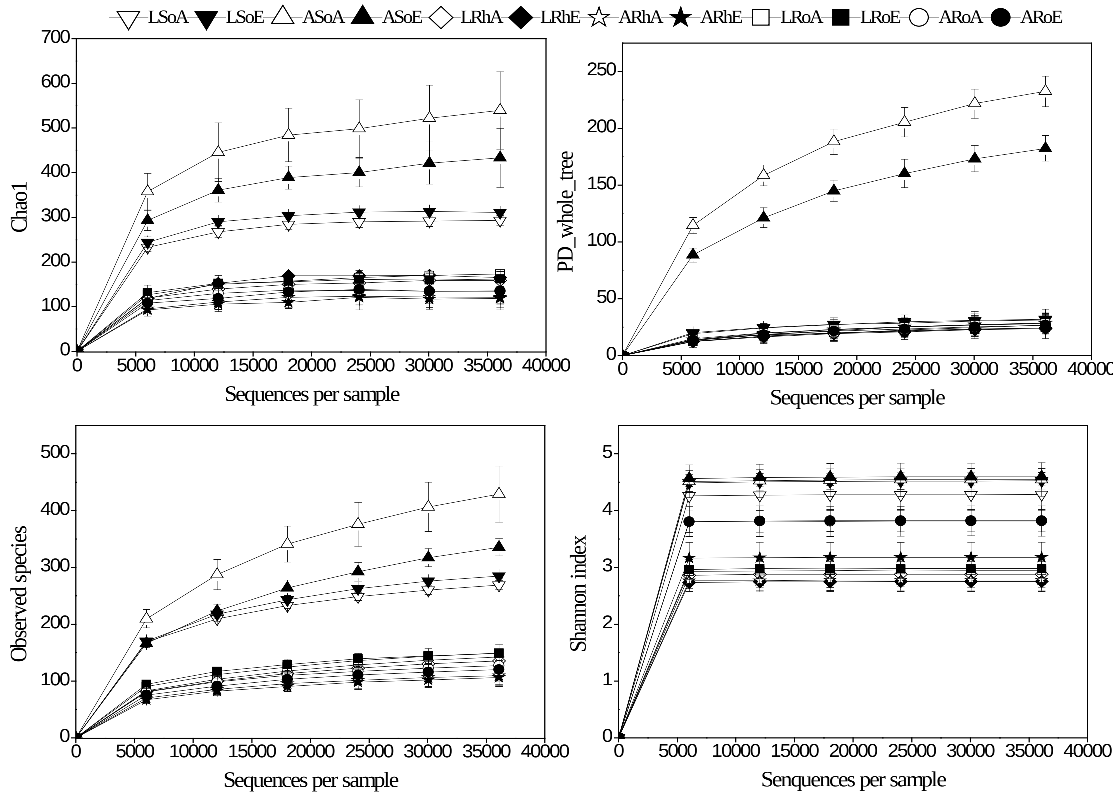

Figure S1. Rarefaction curves of Chao1, Phylogenetic Diversity (PD) index, observed species, and Shannon index for the three compartments in the *C. lasiocarpa* and *C. angustifolia* marshes under ambient and elevated CO_2 concentrations. Vertical bars denote standard deviation of the mean (n = 3). Group names have been selected according to the treatments: L and A denote the *C. lasiocarpa* and *C. angustifolia* marshes, respectively; So, Rh, and Ro indicate the soil, rhizosphere soil, and root compartments, respectively; and A and E represent ambient and elevated CO_2 concentrations, respectively.
